# Publisher’s Note: Update of Journal Title Abbreviation

**DOI:** 10.3390/jox16040154

**Published:** 2026-08-20

**Authors:** Journal of Xenobiotics Editorial Office

**Affiliations:** MDPI AG, Grosspeteranlage 5, 4052 Basel, Switzerland; jox@mdpi.com

Starting with Issue 5 Volume 16, the *Journal of Xenobiotics* will adopt the abbreviation *J. Xenobiotics*, in accordance with ISO 4 standards for the abbreviation of journal titles. The abbreviated-title metadata contained in the PDF and XML files of articles published prior to this change will not be updated retrospectively. Only the journal abbreviation displayed on MDPI’s web-based platforms will be revised to reflect the updated abbreviation.

This update applies only to the ISO4 journal title abbreviation. The journal’s branded short title, *JoX*, remains unchanged and is not affected by this update.

